# Corpus linguistics and clinical psychology: Investigating personification in first-person accounts of voice-hearing

**DOI:** 10.1075/ijcl.21019.col

**Published:** 2022-04-29

**Authors:** Luke Collins, Vaclav Brezina, Zsófia Demjén, Elena Semino, Angela Woods

**Affiliations:** i Linguistics and English Language, Lancaster University; ii Institute of Education, University College London; iii Department of English Studies, Durham University

**Keywords:** health communication, triangulation, collocation, normalisation, personification

## Abstract

Triangulating corpus linguistic approaches with other (linguistic and non-linguistic) approaches enhances “both the rigour of corpus linguistics and its incorporation into all kinds of research” (McEnery & Hardie, 2012:227). Our study investigates an important area of mental health research: the experiences of those who hear voices that others cannot hear, and particularly the ways in which those voices are described as person-like. We apply corpus methods to augment the findings of a qualitative approach to 40 interviews with voice-hearers, whereby each interview was coded as involving ‘minimal’ or ‘complex’ personification of voices. Our analysis provides linguistic evidence in support of the qualitative coding of the interviews, but also goes beyond a binary approach by revealing different *types* and *degrees* of personification of voices, based on how they are referred to and described by voice-hearers. We relate these findings to concepts that inform therapeutic interventions in clinical psychology.

## 1 Introduction

Corpus linguistics as a versatile methodology has been employed in a number of areas of social (e.g. Baker et al., 2013; Taylor & Marchi, 2018; Dayrell et al., 2020) and healthcare (e.g. Crawford et al., 2014; Semino et al., 2017; Baker et al., 2019) research, bringing evidence about patterns of language use to complement evidence from other sources. In the context of healthcare, understanding these patterns provides crucial insights into lived experiences of health and illness.

In this paper, we discuss and exemplify how corpus linguistics can be used in the analysis of data in clinical psychology on the basis of forty semi-structured research interviews with users of ‘Early Intervention in Psychosis’ (EIP) services (Alderson-Day et al., 2020). We apply corpus linguistic methods to a live issue in clinical psychology: the ways in which people who hear voices that others cannot hear – known as ‘voice-hearers’ – perceive those voices as persons. This issue has both theoretical and therapeutic relevance, and corpus linguistics can serve both to triangulate clinical psychological research methods and as a means to expand on their insights regarding the perception and depiction of voices as “persons” in novel ways. Such analysis can help us understand the nuanced experiences of voice-hearers and potentially address them more effectively.

‘Voice-hearing’ refers to auditory perceptual experiences in the absence of external stimulation (Beck & Rector, 2003). In clinical contexts, voice-hearing is associated with psychosis and schizophrenia, as well as other conditions (Zhuo et al., 2019). However not everyone who hears voices is disturbed by them or has a diagnosed mental health problem. In fact, even people seeking help for these experiences represent a heterogenous group, ranging from those who cope well to those who are highly distressed and seek urgent clinical care (Maijer et al., 2017).

One dimension that contributes to this variation in experiences is the extent to which voices are perceived as ‘personified’, i.e. as fully-fledged social actors in their own right (with associated personalities, histories, motivations, and free will), as opposed to being perceived as disembodied words or sounds (Alderson-Day et al., 2020). For example, when asked to describe their experiences, a voicehearer might describe their voice as “just some lad that just chatters on about crap constantly”, while another might be willing and able to say much more, including information about what the voices do and their point of view: “she holds back a lot because she, she knows how like exhausting it can be […] she’ll just sort of try and calm me down […] like a caring person”. These descriptions were provided by Yan and Xander,^1^ two voice-hearers in our data set whose experiences of voices have been respectively classified as involving ‘minimal’ and ‘complex’ personification by the interdisciplinary team at Durham University who interviewed them (see Section 4.1) (Alderson-Day et al., 2020).

Our aims in this paper are to:

- Evaluate corpus linguistic evidence related to the MINIMAL vs. COMPLEX^2^ classification of personification of voices (Alderson-Day et al., 2020)
- Add nuance and detail to the binary classification by comparing systematically the ways in which voices are described in interviews classified as MINIMAL vs. COMPLEX
- Investigate degrees and types of personification in the data

We begin by briefly introducing the insights on personification of voices gained from largely qualitative analyses, including of our dataset specifically (Section 2). We then consider the benefits of applying corpus linguistic methods to analyse interviews with voice-hearers, as well as the process of triangulation – bringing evidence from clinical psychology and corpus linguistics together (Section 3). After introducing our data and methodology (Section 4), we demonstrate how corpus methods can be adapted and utilised for the purposes of the analysis of interviews with voice-hearers (Section 5).

## 2 Approaches to personification of voices in clinical psychology

Social cognitive approaches to voice-hearing within clinical psychology posit voices as “hallucinated social identities” or “internalised social actors”, rather than simply “hallucinated words or sounds” that are not attributed to a clear person-like internal or external source (Bell, 2013:1). Several studies have documented a range of complexities in terms of how ‘person-like’ voices are reported to be (Nayani & David, 1996; Wilkinson & Bell, 2016). Voices may be attributed attitudes, intentions, and different kinds of identities, including proper names. They may also be involved in the kinds of interactions that are typical of social relationships in external interpersonal social contexts (see also Hayward et al., 2011; Hayward et al., 2015), including producing “coherent communicative speech acts” (Bell, 2013: 1). The person-like nature of voices has been reported in studies of both help-seeking and non-help-seeking voice-hearers (Daalman et al., 2011; Kråvik et al., 2015).

Studies of voice-hearing, as an area of mental health research, have drawn on a range of cross- and interdisciplinary approaches, with “rich bodies of literature” in psychiatry, cognitive psychology, anthropology, medical humanities, sociology, philosophy and literary studies (Woods et al., 2014: S247). In thinking about the contribution that the findings of our research can make to therapeutic interventions, we position voice-hearing research as an area of clinical psychology, which has typically relied upon semi-structured interview data and questionnaires (Baumeister et al., 2017), such as the commonly-used Psychotic Symptoms Rating Scale (PSYRATS). Categorizations of the person-like aspects of voices are typically based on descriptions provided by voice-hearers themselves, which have been shown to be separate from measures of severity of psychosis (Alderson-Day et al., 2020). In line with approaches to discursive psychology (Edwards & Potter, 1992), we argue that while first-person accounts do not afford straightforward insights into aspects of thought disorder, investigating such reports – and recognising the interactional contexts in which they are generated – provides key evidence for understanding the lived experience and appraisal of, in this instance, voices. In an online survey of 153 voice-hearers, Woods et al. (2015: 330) found descriptions of the person-like aspects of voices to be among “the most common aspects of voice-hearing” with more ‘characterful’ voices having a greater potential to influence the voice-hearer and thereby amenable to more meaningful engagement. The perception of person-like attributes in the voice has implications for therapeutic interventions that are increasingly characterized by an emphasis on engagement with and making sense of voices within an interpersonal framework (Thomas et al., 2014; Deamer & Hayward, 2018). Differences in the kinds of social actors and relationships with voices experienced by voice-hearers relate to different levels of distress (Wilkinson & Bell, 2016). Understanding voices as persons, therefore, potentially allows voice-hearers to apply strategies they use to navigate everyday social interactions in their relationships and encounters with their voices (Bell, 2013).

In recognition that first-person reports of voice-hearing attest to the perception of person-like agents, clinical psychological studies have developed ways of classifying the personification of voices. For example, Wilkinson and Bell (2016) propose a taxonomy for describing the complexity of social agent representations, ranging from absent agency (i.e. auditory hallucinations that are not vocal, such as clicks or bangs) to internally and externally individualised agency, i.e. agents identifiable by individual characteristics that make them “trackable” over time, with externally individualised agents linked to specific people from the “outside world”.

Alderson-Day et al. (2020) generated their own binary classification of participants’ descriptions of voices, as either MINIMAL or COMPLEX in terms of ‘personification’:

**Table:** 

MINIMAL personification:	The voice has few person-like qualities; is attributed to a person or described as being “like a person” but without further elaboration. Person-like characteristics tend to remain stable over time and follow a single theme (e.g. the voice is “mean”, or a “nasty man”).
COMPLEX personification:	The voice is described as having more than one kind of personlike quality. These may include elaborate descriptions of intentional states (the voice wants/thinks/feels), agency (the voice will “make something happen”), or identity (the voice “comes” from somewhere or has a specific and idiosyncratic ontological status). Complexity is not a simple function of the frequency, quantity or topic of speech, but will typically involve a voice being attributed multiple, qualitatively different person-like qualities (e.g. voice has an identity and multiple mental states) which may vary over time. (Alderson-Day et al., 2020:6)

Alderson-Day et al. (2020:7) deploy this categorisation to facilitate a content and thematic analysis of interviews with 40 voice-hearers, statistically examining key associations with personification and finding that “voices with complex personification stood out as affording companionship and conversation”. Alderson-Day et al.’s (2020) MINIMAL-COMPLEX classification was applied to the dataset that is the basis of our own analysis below. As such, it directly informed our linguistic approach to the investigation of complexity of personification.

These kinds of analyses and categorizations from clinical psychology “fundamentally rely on what is, in effect, manual annotation of patient language”, similarly to other psychometric instruments (Resnik et al., 2014:iii) and language focused analyses are an important part of studies of voice-hearing. For example, Woods et al. (2015:33) argue that linguistic analyses “yield insights into what people who hear voices themselves regard as most important”, De Boer et al. (2016) compared differences in the linguistic structure of voice ‘utterances’ in clinical and non-clinical voice-hearing and Tovar et al. (2019) examined differing use of first- and second-person constructions in people with schizophrenia. Further examples include Fenekou and Georgaca (2010) and Milligan et al. (2013) among others. Nevertheless, linguistic approaches to personification in voice-hearing are rare. One exception is Semino et al. (2021), who use concepts and insights from the literary linguistic study of story-world characters to shed new light on the nature and degree of voices as social agents. In this paper, we go beyond the qualitative approach adopted by Semino et al. (2021) by exploiting the potential of corpus linguistic methods to triangulate the findings of psychological research relying on different methods and ultimately to contribute to creating “scalable, inexpensive screening measures or risk assessments that may be administered by a wider variety of healthcare professionals in a broad range of contexts” (Resnik et al., 2014: iii). One existing tool that offers quantitative linguistic analysis of psychological phenomena such as trauma, bereavement, deception etc. is the *Linguistic Inquiry and Word Count* (LIWC) text analysis program (Pennebaker et al., 2015), which automatically categorises language data according to pre-defined categories. Our approach required a specific focus on references to voices and a classification based on part-of-speech, prior to an inductive thematic grouping of terms according to how they were used in the context of the interview. Further details of our approach are provided in Section 3.

We discuss the implications of our findings both for “enhancing both the rigour of corpus linguistics and its incorporation into all kinds of research, both linguistic and non-linguistic” (McEnery & Hardie, 2012:227) and for the nature and characteristics of voices as personified social actors in clinical psychology. Finally, by approaching the attribution of person-like qualities to voices as a matter of degree, we contribute to debates in clinical psychology around the extent to which experiences related to psychosis can (or should) be considered on a single continuum from non-clinical to clinical (Baumeister et al., 2017; Powers et al., 2017; Collins et al., 2020).

## 3 Corpus linguistics and our approach to triangulation

Corpus linguistics allows us both to quantify the occurrence of a target word or phrase and to discern linguistic meaning in context through observing patterns of word occurrence via concordance analysis (Brezina & McEnery, 2020). This kind of evidence has been shown to complement the findings from other methods, such as discourse analysis and psycholinguistics (Egbert & Baker, 2020a). Corpus linguistics thus enables us to contribute to a growing body of research, which triangulates the evidence obtained by using multiple research methods (e.g. Egbert & Baker, 2020a; Thurmond, 2001). In this paper, we focus on the contribution of corpus linguistics to a particular concern in clinical psychology, the personification of voices that others cannot hear.^3^ This involves careful consideration of the most suitable techniques from the corpus linguist’s toolbox, as well as the necessary adaptation of these methods to the specific context. In this section, we discuss the following key methodological adaptations:

– Linguistic operationalization of the clinical psychology approach to personification
– Corpus annotation of features relevant to such personification
– The role of text length and the normalisation of frequencies for evaluating complexity of personification in relation to descriptions of voices

Let us start with the basics. The first step in any analysis is the operationalization of the concept: “a clear theoretically grounded specification of what the research wants to investigate” (Brezina, 2018a:264). In our specific context, concepts in clinical psychology, such as voice, personification and complexity need to be operationalised in terms of linguistic features which are associated with these concepts in the interviews. Appropriate operationalisation of the key concepts grounded in the social psychological literature is essential for relating corpus linguistic analysis to more qualitative accounts provided by clinical psychology, and for the success of the whole triangulation enterprise (Heale & Forbes, 2013).

Thurmond (2001) distinguishes four types of triangulation: (i) data triangulation, (ii) investigator triangulation, (iii) methodologic triangulation and (iv) theoretical triangulation. These involve (i) combining multiple data sources; (ii) employing multiple investigators with different perspectives to counterbalance potential bias; (iii) using a range of methods on the same dataset; and (iv) grounding the research in different theoretical frameworks. Types (i)–(iii) are available in the collaboration between clinical psychology and corpus linguistics; theoretical triangulation does not apply, since although corpus linguistics is predicated on certain theoretical positions, it principally represents a methodological approach (McEnery & Hardie, 2012) that is not attached to a particular theory. In our case study, we primarily demonstrate methodologic triangulation (explicitly), which also involves investigator triangulation.

While automated annotation of corpora for part-of-speech (e.g. Leech et al., 1994; Schmid, 1994) or semantic categories (Rayson, 2008) can be helpful in corpus-based studies of clinical psychology data (e.g. Collins et al., 2020), the understanding of specific constructs in clinical psychology requires knowledge of broader contexts and hence the adoption of manual coding. Manual coding can be aided by other levels of annotation (part-of-speech, semantic categories) and can be facilitated by automatic searching for key terms. A crucial linguistic point to make here is the fact that dealing with interview data requires analysis at the level of pragmatics, encompassing contextual meanings and inferences. Aijmer and Rühlemann (2015) consider three options, which can be combined, in order to identify a *locus* of pragmatic meaning:

1. Using as search terms words/structures that carry pragmatic meaning (e.g. swearwords, discourse markers, politeness markers, stance (position) markers)
2. Delimiting the linguistic settings with a specific pragmatic meaning and investigating linguistic features, which occur in these settings
3. Employing pragmatic tagging of corpora to aid the searching/interpretation

In this study, we focus on selected markers of personification. Our starting points were the notion of voices and definitions of personification applied to our data in the manual coding completed by Alderson-Day et al. (2020), outlined in Section 2. Subsequently, we focused on a small number of elements that provided the most explicit realisations of those aspects of personification in terms that could be queried through corpus analysis tools. Specifically, three relevant linguistic categories were identified in the data: (1) references to voices to operationalize the clinical psychological concept of voice, (2) explicit descriptions of voices’ qualities and characteristics, and (3) descriptions of activities and processes that voices are involved in. Categories 2 and 3 relied on the definitions inherent in Alderson-Day et al.’s (2020) MINIMAL-COMPLEX classification of personification and therefore operationalise this construct for our purposes. As ‘MINIMAL personification’ was identified in the absence of any evidence for complexity (“without further elaboration”), we focus on the definition of ‘COMPLEX personification’ as a ”positive” indicator (i.e. presence, rather than absence). We operationalised “having more than one kind of person-like quality” in the definition of complex personification, along with ”elaborate descriptions of intentional states (the voice wants/thinks/feels)” and “agency (the voice will “make something happen”)” as the following three “language components” of personification:

- The range of terms used to refer to the voice (hereafter, ‘Voice labels’)
- The adjective collocates of those references e.g. *aggressive*, *young*, *tall*
- The verb collocates of the references e.g. *talk*, *mean*, *control*

A unique identifier (_VOICE) was manually added^4^ to the annotation generated by the part-of-speech tagger for nominal and pronominal references to voices. References were typically pronouns (*it*, *she*, *they*) and nouns (*voices*, *shadow*, *Roxy*), though were also found in determiners (*this*, *some*, *which*), verbs as gerunds (*commenting, whispering*), numbers (*the first one*) and adjectives (*there’s one good and one bad*). The tagging allowed us to overcome the challenge identified by, for example, Hardstaff (2015), in missing out on a large number of anaphoric and cataphoric references to a subject.

Finally, we needed to consider one last adaptation: the process of normalisation appropriate to our context. Normalisation is one of the fundamental procedures of the corpus approach. It is outlined by Biber et al. (1998), for example, as one of their “methodological boxes”, alongside aspects of corpus design, statistical tests and units of analysis. In a typical corpus linguistic study, normalisation involves the computation of the relative frequencies of the linguistic features under investigation, mainly to allow for a fair comparison between texts and (sub)corpora of different length (Brezina, 2018b:43). The underlying assumption behind normalisation is that, overall, there is approximately an equal opportunity for a linguistic feature to occur in any stretch of text so that, as Biber et al. (1998:263) explain, there are more opportunities in a longer text for a feature to occur. Furthermore, there is a linear relationship between the text length (overall number of tokens) and the frequency of any linguistic feature. With large datasets, which are typically used in corpus linguistics, this is a fair assumption. However, when the focus is on individual speakers such as participants in clinical psychology interviews, where the topics and content of the text do not emerge organically, but are guided in a targeted way, the picture is slightly more complicated. For the purposes of analysing complexity of personification in our self-reports, therefore, we needed to re-think the normalisation process.

It has been pointed out by Buttery et al. (2012), in the context of learner language, that the opportunity of use of different linguistic features may differ with text length when the performance of individual speakers is considered. With respect tothe interviews with voice-hearers analysed here, the length of the interviews is an important indicator of the complexity of the narrative, which in turn has important diagnostic implications (Alderson-Day et al. 2020). Also, when measuring the variety of the types of different linguistic features to establish the complexity of the target linguistic features, simple normalisation of frequencies is not appropriate. This is because of the well-established effect of text length on the type/token ratio: the longer the text, the smaller the type/token ratio due to repetition and lexical recycling (Covington & McFall, 2010; Brezina, 2018b: 57). The voice-hearer participants were all asked the same base interview questions. However, their responses ranged from 1138 to 14475 tokens (inclusive of all fillers, discourse particles etc.). Recognising that the length of the response would affect the frequency and variety of types in a given interview, we wanted to acknowledge the importance of the number of tokens in each interview as a potential indicator of complexity,^5^ whilst minimising the impact that the text length would have on our tabulations for our language component types. We therefore decided to (i) include interview length as a fourth language component in our analysis; and (ii) normalise the frequencies for the other language components of our investigation of complexity of personification (see 4.2) to control for the effects of text length.

We conducted our corpus analysis using *#LancsBox* (Brezina et al., 2015), which has an automatic splitter among its pre-processing tools. We used this tool to split each interview into 500-word chunks. We subsequently counted the number of types for each language component in each chunk and calculated an average for each participant. Since the splitter is applied before a tokeniser and since participant files were typically not a round multiple of 500, chunks of 500 words were only approximate to 500 tokens. As such, type counts were also normalised to a value per 100 tokens. For instance, an interview with Nina generated 4111 tokens of speech, which we split into nine chunks: eight chunks of approximately 500 tokens plus one chunk of the remaining tokens. The number of Voice labels in each chunk was 12, 15, 7, 6, 4, 8, 5, 10 and 7, which we converted to a relative frequency of per 100 words. The resulting average was a value of 1.82 for Voice labels in Nina’s interview. All of our reported values reflect the average number of types per 500 tokens, normalised to per 100 tokens. This process helped us to minimise the effect of the text length on other linguistic components, whilst accounting for the fact that certain topics that might prompt references to voices could appear at different stages in the interview. Since the definition of complexity of personification highlighted the importance of exhibiting a range of qualities, our frequency analysis is based on the number of types, rather than tokens, but we also consider the total number of tokens produced as a diagnostically relevant feature.

## 4 Data and methods

In this section we explain our selection of linguistic components for identifying and evaluating the complexity of the personification of voices, before outlining the procedures by which we calculated measures of complexity. Firstly, we introduce the interview data collected from voice-hearers.

### 4.1 Data

Our dataset consists of 40 semi-structured interviews with voice-hearers using “Early Intervention in Psychosis” services in the North East of England, conducted as part of the Wellcome-funded Hearing the Voice project at Durham University (https://hearingthevoice.org). Study participants were all (i) aged 16–65; (ii) heard voices at least once a week for a month; (iii) fluent English speakers; (iv) had normal or corrected-to-normal vision; and, (v) were in the first nine months of using EIP services. They provided written consent, including for the reproduction of direct quotes from their interviews. All procedures were approved by a local NHS Research Ethics Committee.

Interviews typically lasted one hour (ranging from 24–103 minutes). The Hearing the Voice Phenomenology Interview (Alderson-Day et al., 2020) included questions related to: how participants would describe their experiences; the qualities and content of the voice-hearing experience; whether the voices have their own character or personality; the onset of voice-hearing; changes in the experience over time; and participants’ beliefs about/understanding of the experience. The interviews were transcribed and manually coded by the Hearing the Voice team for a number of clinically relevant phenomena (see Alderson-Day et al., 2020), including, as we have mentioned, the binary classification of MINIMAL or COMPLEX, according to the definitions provided in Section 2. While it was possible that participants could report voices that, separately, could be assessed as minimal, or complex, they were exclusively assigned to one of the classifications (i.e. someone reporting a combination of minimal and complex personified voices would be coded as COMPLEX). Twenty-four out of the 40 interviews were coded as MINIMAL and 16 as COMPLEX for personification.

### 4.2 Linguistic operationalization of complexity of voice personification

For the purposes of our analysis, the interviewer’s questions were removed from the files, which left us with a dataset of 205941 tokens and 7655 types across the 40 interviews. Our analysis centres on specific references to voices in our data and we focus on three language components to capture different specific aspects of personification, as outlined above: terms used to refer to voices (Voice labels), and adjective and verb collocates of these references. In addition, we also considered the length of the participant contributions, which we discuss in terms of its correspondence with complexity.

As noted above, this study has three interconnected aims:

- Evaluate corpus linguistic evidence related to the MINIMAL vs. COMPLEX classification of personification of voices
- Add nuance and detail to the binary classification by comparing systematically the ways in which voices are described in interviews classified as MINIMAL vs. COMPLEX
- Investigate degrees and types of personification in the data

We address the first aim by looking at the group- and individual-level rankings based on frequencies of types for our language components, along with the length of participant contributions (i.e. number of tokens). With respect to our second aim, we report on differences in the use of particular types across the two groups of interviews. Finally, we address our third aim using four individual cases to demonstrate how particular types associated with complexity operate in combination to reflect varying degrees of complexity of personification.

#### 4.2.1 Procedure

The data was manually annotated for references to voices to enable automated corpus queries and the identification of adjective and verb collocates. This Voice label was used as the node for the identification of verb and adjective collocates via the GraphColl tool in *#LancsBox*^6^ (Brezina et al., 2015). Since we intended to capture the full range of collocates that were used alongside references to the voices, rather than investigate the strength or exclusivity of collocates, absolute frequency was used to identify collocates, with a minimum frequency threshold of 1. We subsequently normalised these frequencies according to the procedure described in Section 3, to facilitate a comparison of cases at the individual and group levels. We used a collocational span of 3 tokens either side of the node to identify adjective collocates and a collocational span of 3 tokens to the right of the node for verb collocates. While these settings would not capture every description of the voice(s) and what they are reported as doing (e.g. using a passive construction), a manual check of a sample of the results from other possible collocational spans showed that they were optimal for the precision and recall of characteristics and processes directly attributable to a voice.

We compared the interview responses coded as MINIMAL with those coded as COMPLEX with the aim of identifying which language components (Voice labels, adjective collocate types, verb collocate types, and text length, i.e. tokens) that were more characteristic of either the MINIMAL or COMPLEX cases and for mapping out a scale of complexity. Specifically, the following steps were taken:

1. Statistical comparison of the MINIMAL and COMPLEX cases: We performed an independent samples t-test comparing interviews originally coded as MINIMAL and COMPLEX at the group level with respect to each of our language components. This enabled us to consider the validity and significance of the complexity groups in relation to our language components.
2. Distribution of MINIMAL and COMPLEX cases: We generated rank lists of individual normalised frequency values for each language component to investigate the distribution of MINIMAL and COMPLEX cases. This allowed us to assess the variation within and across MINIMAL and COMPLEX interviews, on the basis of the relative frequency of our language components.
3. Categorising types: We compared the types of Voice labels, adjective collocates and verb collocates found in a high proportion of interviews in each group. This enabled us to identify if particular types were associated more with MINIMAL or COMPLEX cases. Given the number and variety of individual types, we grouped semantically-related types to identify patterns. For example, with respect to Voice labels, we were able to distinguish the more “person-like” terms *bloke*, *guy*, *people*, *woman* (grouped as “Persons”) from “non-humans” like *angel*, *demon*, *spirit* (grouped as “Supernatural”) and *birds*, *flies*, *raccoon* (grouped as “Animals”). These groupings were generated inductively from the range observed in the data and based on the meaning of the types determined by examining concordance lines and categorising according to their most frequent usage.
4. Individual cases: Using the rank frequency lists generated in step 2, we selected a small number of cases for more detailed investigation of the upper and lower limits of the complexity groupings, giving us a view of the range within the cohort. Specifically, we selected:
  - A high-ranking COMPLEX case
  - A low-ranking MINIMAL case
  - A high-ranking MINIMAL case
  - A low-ranking COMPLEX case

We present evidence for different ways in which Voice labels and collocate types are used in different types of cases.

## 5 Results and discussion

This section of the paper will be divided according to our three aims. First, we address the corpus linguistic evidence related to the clinical coding of the interviews as MINIMAL or COMPLEX personification in terms of group level differences and individual rankings based on the frequency of our language components (5.1). We then report the relevant differences in the use of particular types favoured by participants in the respective complexity groups to show the detailed characteristics of descriptions of voices classified as MINIMAL vs. COMPLEX (5.2). Finally, we refer to the interviews of four individual cases to demonstrate how our language components and the use of types associated with complexity operate in combination to reflect varying *degrees* of complexity of personification (5.3). This allows us to go beyond the binary coding and to begin to map out a complexity scale, or continuum, of personification of voices (see Baumeister et al., 2017; Powers et al., 2017; Collins et al., 2020).

### 5.1 Corpus linguistic evidence related to the MINIMAL/COMPLEX binary classification

At the group level of analysis, we found meaningful differences between interviews coded as MINIMAL and those coded as COMPLEX across our four language components. As shown in Table 1, the independent samples t-test, carried out using the normalised values for each of the language components, confirmed statistically significant differences between the groups with respect to number of Voice label types, adjective collocate types, verb collocate types and tokens. In each case, we observed, on average, higher values for the complex personification group than the minimal personification group. The observed standardized effect size (Cohen’s *d*) was also large in each case^7^ and non-overlapping confidence intervals for two groups suggest more generally that there is a meaningful difference between the two groups in terms of these four language components. Our findings are therefore convergent (Egbert & Baker, 2020b) with those of the qualitative coding scheme for complexity carried out by the Hearing the Voice team (Alderson-Day et al., 2020).

In Table 2, interviews are ranked by the relative, normalised values for the number of types of our language components (for Length, this is simply the number of tokens). Interviews are labelled according to their pseudonym and complexity coding (_M indicating MINIMAL, _C indicating COMPLEX). The general distribution of interviews in Table 2 is also broadly convergent with the complexity coding: cases of COMPLEX personification tend to cluster at the top of the lists (e.g. Leah_C, Page_C, Xander_C), while cases of MINIMAL personification tend to cluster at the bottom (e.g. Brad_M, Dawn_M, Harry_M). This again suggests convergence with Alderson-Day et al.’s (2020) manual coding.

However, we can also see from this ranking that there are overlaps between the two groups: there is a mix of COMPLEX and MINIMAL cases in the middle of the rank lists. This suggests that there is no clear threshold for complexity, or clear separation between the two groups on the basis of frequency. We can also see from these lists that there appears to be quite a large variation within both the MINIMAL and the COMPLEX groupings. For example, Carl_M appears towards the top of three out of four of the rank lists, indicating that he used a comparable number of Voice label types, adjective collocate types and verb collocate types to cases coded as COMPLEX. Interestingly, he produced this range of types in one of the shortest interview responses (he is ranked 38th for token length). Conversely, Violet_C tends to appear towards the bottom of the lists, suggesting a relatively restricted number of types that is more comparable with cases coded as MINIMAL. Furthermore, the relatively high type counts for Carl_M and relatively low type counts for Violet_C begin to suggest that other (qualitative) differences are likely to have contributed to their complexity coding. The next stage of our analysis offered some indication that the use of particular types, in addition to the number of types, constitutes one such difference.

### 5.2 Linguistic differences between MINIMAL vs. COMPLEX descriptions of voices

To add nuance to the binary coding of the interviews (aim “b”), we looked at the dispersion of particular types within each group. Here, we focus on types that are used in a high proportion of interviews within one complexity group and that offer a point of contrast with the other complexity group (i.e. MINIMAL vs COMPLEX). We mainly discuss differences in terms of semantically-related types, rather than individual type level differences, which are presented according to the groupings we developed inductively to account for the range of terms used across the data. This allows us to better account for the variety of ways in which similar concepts relating to personification might be articulated. We provide examples of the types that made up our semantic groupings before discussing relevant differences with respect to our language components (excluding token length).

#### 5.2.1 Voice label types

We grouped Voice label types (with examples of constituent types in italics) as follows:

– Persons: e.g. *girl*, *man*, *people*, *somebody*
– Names: *David*, *May*, *Noah*, *Roxy*
– Functional/Occupational terms: *bully*, *criminals*, *officer*
– Social relationship and familial terms: *dad*, *ex-girlfriend*, *friend*, *grandma*
– Pronouns:
  – First person: *I*, *me*, *us*, *we*
  – Second person: *you*, *your*
  – Third – Feminine: *her*, *herself*, *she*
  – Third – Masculine: *he*,*him*, *his*
  – Third – Neuter: *it*, *them*, *they*
  – Demonstrative: *these*, *what*, *which*, *who*
– Body parts: *face*, *hands*, *heads*, *mouth*
– Non-humans:
  – Supernatural: *angel*, *demon*, *God*, *spirit*
  – Animals: *birds*, *flies*, *racoon*
  – Objects: *bombs*, *cars*, *keys*, *tree*
– Speech acts and communication: *accusations*, *comments*, *talking*, *threats*
– Noises:*click*, *crashing*, *scratching*, *wailing*
– Message content: *messages*, *phrases*, *sentences*, *words*
– Visual elements: *colours, flashing, image, shadow*
– Actions: *bouncing*, *movement*, *vibrating*
– Felt: *brushes*, *sensation*, *touch*
– Taste and smell: *bread, manure, popcorn, smell*
– Scenario: *plots*, *scenario*, *situation*
– Cognition: *memory*, *opinions*, *thoughts*
– Number: *all*, *both*, *four*, *majority*
– Undetermined: *something*, *stuff*, *things*, *whatever*

Overall, we observed a greater number of individual Voice label types in the 16 COMPLEX cases (305) compared with the 24 MINIMAL cases (183), with 102 types appearing in both. At the group level, we found that a higher proportion of COMPLEX cases (8, 50%) used Names when referring to their voices, compared with MINIMAL cases (5, 20.8%). Similarly, 7 (43.8%) COMPLEX cases featured “Social relationships and familial terms” (compared with 6 (24%) MINIMAL cases), for example in identifying a voice as a deceased relative. We also observed a higher proportion of first- and second-person pronouns in reference to the voices in COMPLEX cases (14 (87.5%) and 11 (68.8%) respectively, compared with 12 (50%) and 10 (41.7%) MINIMAL cases).

We interpret having a name and social relationships as directly indicative of personification. The differences in the use of pronouns between the groups also reflect a greater potential for conversation or interaction between the voice and the voice-hearer in COMPLEX cases. First-person pronouns often occurred in instances of direct speech reporting that assumed the viewpoint of the voice e.g. *it was saying, ‘I’m here’* (Zara_C), and voices could also align themselves with the voice-hearer: *why don’t we go outside?* (Eric_C). Among 153 instances of a first-person pronoun tagged as a Voice label, 118 (77.1%) were used by COMPLEX case participants. The use of second-person pronouns indicated that the voice-hearer could speak to and address the voice directly, for example *you don’t tell the truth* (Eric_C). Forty-seven (66.2%) of the occurrences of second-person pronouns as Voice labels were found in COMPLEX case interviews.

#### 5.2.2 Adjective collocate types

With regard to adjective collocate types, the semantic groupings were as follows:

– Demographics:
  – Gender and sexual identity: e.g. *feminine*, *gay*, *lesbian*, *male*
  – Age: *childlike*, *old*, *young*
  – Ethnicity/region: *American*, *regional*, *scouse*
– Personality traits, mood and demeanour:
  – Personality traits: *confident*, *friendly*, *impulsive*, *mischievous*, *nasty*
  – Demeanour: *cheery*, *commanding*, *gentle*, *harsh*
  – Emotional states: *angry*, *annoyed*, *happy*, *scared*
– Beliefs and perspective: *evil*, *homophobic*, *hypochondriac*
– Ability: *capable*, *clever*, *powerful*, *useless*
– Non-human: *demonic*, *inhuman*
– Perceptible qualities : *clear*, *faint*, *invisible*, *prominent*
  – Auditory: *deep*, *loud*, *low*, *squeaky*
  – Visual: *angular*,*big*, *black*, *dark*
  – Identifiable: *distinct*, *familiar*, *particular*
– Location: *above*, *distant*, *internal*, *outside*
– Time and duration: *brief*, *constant*, *frequent*, *sudden*

Similarly to Voice label types, there were more unique adjective collocate types in the 16 COMPLEX case interviews (318), than in 24 MINIMAL cases (229), with 126 shared between the two groups. Our groupings enabled us to assess the prevalence of patterns of associated features that manifested in different ways in individual cases. For instance, there were 57 instances of *angry* across 10 cases in this grouping, with 93 occurrences overall of terms referring to emotional states across the cohort.

At the semantic group level, adjective collocate types grouped as Demographics were more evident in COMPLEX cases. These adjectives strongly implied person-like qualities in the sense of gender, age and ethnicity associated with voices. For example, *male* appeared as an adjective collocate in 9 (56.3%) COMPLEX cases, but only one (4.2%) MINIMAL case; and *female* appeared in 8 (50%) COMPLEX cases but only 4 (16.7%) MINIMAL cases. Furthermore, the MINIMAL case participant that used both stated that *It was hard to kinda define it as a male or female voice* (Anthony_M).^8^ In COMPLEX cases, Demographic types also co-occur to further distinguish a voice, as in *the youngest male voice* (Jade_C).

While it was common for participants from both complexity groups to refer to negative voice characteristics (in part, accounting for their enrolment with clinical services), we observed a higher frequency of directly contrastive positive characteristics among the COMPLEX cases. Thirteen (81.3%) COMPLEX cases referred to voices with both negative descriptors, e.g. *bad*, *negative*, *nasty* and/or *distressing* and contrasting positive descriptors *good*, *positive*, *nice* and/or *comforting*. In some instances, this dual characterisation was extended to account for intermediary positions; for example, Nina_C reported that *there are some that are nice, some that are not nice, some of them are neutral*. In contrast, only 4 (16.7%) MINIMAL cases referred to contrastive negative and positive traits and these were largely reported in terms of two contrasting voices: Liam_M referred to *a good one and a bad one*; and Ryan_M refers to a *good cop, bad cop* dynamic, wherein while the “good cop” Noah *is still negative, he’s more compassionate*. In COMPLEX interviews, while it was also common to attribute contrasting qualities to different voices, participants more often referred to one voice in terms of multiple (contrasting) qualities, for example, Leah_C reported that *He does good things […] but he’s vicious*. This suggests that complexity has the potential to afford more positive experiences, which in turn has implications for relational therapies (Thomas et al.,2014), in which voice-hearers explore opportunities to engage with and develop their relationships with their voices.

#### 5.2.3 Verb collocate types

We grouped verb collocate types as follows:

– Communicative actions and noises:
  – Speech acts: e.g. *criticise*, *offer*, *question*, *warn*
  – Speech sounds: *say*, *tell*, *talk*, *shout*
  – Dialogue/turn-taking: *argue*, *respond*, *answer*
  – Non-speech noises: *knock*, *laugh*, *cry*
– Perceptual and cognitive verbs:
  – Perceptual: *hear*, *see*, *find*, *recognise*
  – Cognitive: *know*, *think*, *understand*
– Action: *make*, *use*, *take*, *control*
– Movement: *walk*, *move*, *follow*
– Occurrence: *start*, *happen*, *disappear*
– Relational: *be*, *got*, *seem*
– Modal: *can*, *would*, *might*

As with our two previous language components, we again observed a wider range of individual verb collocate types (347) in the 16 COMPLEX cases than in the 24 MINIMAL cases (283), with 167 verb collocate types appearing in interviews from both groups.

At the semantic group level, many of the verb collocate types – for both MINIMAL and COMPLEX cases – fell into Communicative actions and noises, consistent with the framing of voices in auditory-verbal terms. However, a greater proportion of COMPLEX cases (10, 62.5%) used types in the Dialogue/turn-taking sub-group, than MINIMAL cases (4, 16.7%), indicating that such spoken interactions were more characteristic of COMPLEX cases. For example, Grace_C reported that *they respond sometimes*, Eric_C reported that *she’ll answer, but I need to talk to her first*, and Olivia_C described how her voices *respond* to people in her external world, who are talking to her. Furthermore, the use of these types in MINIMAL cases was often restricted to interactions between voices, for example Sean_M reported overhearing *a man and a woman arguing*.

Linking directly to Alderson-Day et al.’s (2020) definition of complexity, we found indications of ‘intentional states’ in the use of the verb collocate type *want* and the capacity to “make something happen” in the way that the types *make* and *stop* were used. In each case, a greater proportion of COMPLEX interviews used these types compared with MINIMAL cases:

– Fourteen (87.5%) COMPLEX cases referred to voices *want*[*ing*], compared with 11 (45.8%) MINIMAL cases.
– Fifteen (94%) COMPLEX cases used *make*, compared with 13 (54%) MINIMAL cases.
– Twelve (75%) COMPLEX cases used *stop*, compared with 8 (33%) MINIMAL cases.

In the cases of *make* and *stop* we also observed qualitative differences in how these types were used across COMPLEX and MINIMAL cases: while voices in MINIMAL cases were described as simply making *noises* and *racket* (Kate_M), or not making *sense* (Matt_M), in COMPLEX cases *make* was used to describe a transitive process in which the voice *makes* the hearer do or feel certain things. For example, Olivia_C reported that while *they make you unsure of things*, some voices also *made me feel I’m not alone*. Similarly, *stop* in MINIMAL interviews referred to the voices simply *stop*[*ping*], whereas in COMPLEX cases the voice was reported to stop the voice-hearer from doing certain things. For example, Kath_C reported that her voice *tries to stop us from going places* and Dan_C explained that *They’ve stopped me from doing so much*.

The findings from this stage of the analysis provided a basis on which to evaluate complexity of personification with respect to the quality of types, in addition to the relative frequency of types tabulated in previous stages. In the next section, we combine these aspects, and the different language components to investigate degrees and types of personification in individual cases across complexity groups to begin to map out a scale of complexity (aim “c”).

### 5.3 Exploring degrees of complexity through individual cases

Our findings from Section 5.1 showed that, for each of our four language components, complexly personified cases clustered at the top of the ranks, and minimally personified ones clustered at the bottom (Table 2). However, there was overlap between the two groups in the middle of the ranks and large variation within the groups, suggesting continuity between minimal and complex cases along a scale of complexity of personification. In addition, we found that certain participants tended to occupy broadly similar positions in each of the lists based on the normalised rates of Voice labels, verb collocate types, adjective collocate types and total number of tokens in their interviews. This provided an empirical basis on which to select a sample of interviews for more in-depth analysis, which is often a problematic methodological decision. We selected four individuals based on their average position in the rank lists from Section 5.1:

– Brad_M whose average rank list position was 34.5 and therefore the lowest
– Leah_C, whose average rank position was 4.5 and therefore the highest
– Violet_C, whose average rank position was 27.25 and therefore the lowest of the COMPLEX cases
– Carl_M, whose average rank position was 13.25 and therefore the highest of the MINIMAL cases

By applying to each individual the analysis of Voice labels and collocate types, we can evaluate the correspondence between our language components and the complexity coding allocated by the Hearing the Voice team, and begin to differentiate degrees of complexity within and across the groups. In this way, we expand the binary classification towards a scale.

#### 5.3.1 The opposite ends of a scale of complexity of personification

Comparing the two individuals at the “top” and “bottom” of this prospective scale shows a clear contrast between MINIMAL and COMPLEX. As the highest-ranking participant (on average), the complexity of personhood indicated in Leah_C’s case is – in part – evident in that she used 79 different Voice label types, 64 different adjective collocate types and 105 different verb collocate types in reference to her voices. In contrast, the lowest-ranking participant Brad_M, used 28 different Voice label types, 17 different adjective collocate types and 27 different verb collocate types and in both cases, this is consistent with their complexity coding.

Looking more closely at Brad_M’s report, we found that voices were described as doing a relatively narrow range of activities. Most verb collocates simply reflected their existence (*they’re normally quite negative, that’s happened twice*) and auditory verbal nature of the experience (they *say*, *call*, *shout*, *sound*, *speak*, *talk*). Person-like qualities were primarily suggested in the association of the voices with people Brad_M knows in his external world: *I can hear people trying to talk to us, people that I know, that I’m close to, friends, family or something*. Otherwise, he mainly referred to his experiences as inanimate entities (*messages*, *things*, *shadows*, *cars*, *it*) or as an undifferentiated collective (*they*). Despite the low number of types and basic kinds of (verbal) activity attributed to the voices – both of which are consistent with a MINIMAL coding – some degree of personification was indicated in the knowledge and authority attributed to voices. Brad_M reported *I think they think they know what’s best for me* and they were also attributed more tangible actions: *You can feel {.} people slapping you or like {.} touching your back*. This suggests that, at least in this dataset, even an interview that scores lowest across our language components still includes some element of personification.

In contrast, among the numerous types used by Leah_C we unsurprisingly found a wider variety of actions attributed to the voices, and many of these suggested a high degree of personhood. In line with other COMPLEX cases, Leah_C discussed the potential for dialogue with the voices (*I don’t tell them they’re wrong, they tell me I’m wrong*), including referring to them with second-person pronouns: *I still speak to them now. But how do you go up to these people and say, I hear you!*. Linking directly to the definitions for complexity, Leah_C described her voices as having the capacity to “make things happen” in that they *moved us off the quayside*, *sent us on various tasks* and *brought us messages*. Furthermore, the voices carry intentional states and Leah_C assumed their point of view when she quoted them as saying, *we’re going to punish you today*. Leah_C reported that her voices have explicit wants (*do whatever he wants us to do again; we want you to die*), expectations (*I wasn’t expecting you, she says, I was expecting your mother*), and needs (*they don’t need little clicks and stuff like that, he needs clicks and stuff as well*).

The ways in which Leah describes and refers to her voices also demonstrate a wide range of person-like qualities, including physical characteristics (e.g. *big*, *black*, *long*, *round*); moods, feelings, traits and emotions we usually only associate with humans (*ashamed*, *sorry*, *clever*); along with a social hierarchy: *it’s way more powerful than any of the others*. Furthermore, individual voices were attributed contrasting traits: *He does good things, he saves people but he’s, he’s he’s vicious*. While the voices manifested in a variety of entities i.e. human (e.g. *gypsy*), non-human (e.g. *things*; *shadows*), and supernatural (e.g. *angels*, *spirits*, *demons*), they exhibited a range of human characteristics, such as specific names (e.g. *Loki*, *Michael*, *Gabriel*), kinship relationships (e.g. *daughter*, *grandma*), gender (*boy*, *girl*), age (*old)*, sexuality (*she’s a lesbian*), and social relationships (*friends*) that could be independent of Leah_C: *I call them married because it’s as if they’re like in cahoots*. In addition, Leah_C is not always able to predict or interpret what her voices do and this corresponds with Semino et al.’s (2021) discussion of personification, contributing to the voices being perceived as more rounded social agents, or more like “real people”.

Overall, in Brad_M and Leah_C, we found examples of interviews located at distinctly contrastive ends of a prospective complexity scale. In the remaining two cases, we discuss interviews whose position in the rank lists might seem to conflict with their complexity coding. In examining their references to voices in context, we point to other kinds of evidence for their complexity coding and link this to the idea of degrees or scales of personification.

#### 5.3.2 Less clear-cut cases and the middle of a scale of complexity of personification

Referring back to the rank lists from Section 5.1, the normalised values for Voice label types, adjective collocate types and verb collocate types for Violet_C are drastically different to Leah_C, despite both being coded as COMPLEX. Violet_C’s ranks mostly put her interview in the midst of the MINIMAL group. Among the types used by Violet_C, we find evidence for personification in that her voices have names, gender markers (*female*, *male*, *man*), can modulate their speech (*whisper*, *shout*), and perform actions beyond speaking: e.g. *grab*, *they can just pick up things*. Violet_C also described her voices in terms of personal characteristics such as *honest* and *annoyed*. Consistent with those cases coded as COMPLEX, the voices also have the capacity to *make* Violet_C feel particular ways: *sometimes he can make us cry, they make us feel, like Michael makes us feel angry and confused, Margaret makes us feel like happy*. The voices are also reported to have wishes, that Violet has difficulty resisting (*It was really upsetting because I didn’t want to be the person that Michael wanted us to be*), and are described in contrasting terms: *I’ve had bad voices and good voices, like nice ones and bad; But one’s nasty and a bully and the other one’s nice*. However, unlike Leah_C’s report, these contrasting qualities are divided between voices, rather than attributed to one voice. When considered alongside the fact that Violet_C does not report interactions with her voices, or any social relationships independent of the voice-hearer, we can say that Violet_C’s report does not exhibit the same degree of complexity relating to personification as documented in Leah_C’s report. Nevertheless, the examples of the types reported here are consistent with other COMPLEX cases and the definitions that informed the complexity coding.

A counterpart to the example of Violet_C, is Carl_M’s position in the rank lists for Voice label types, adjective collocate types and verb collocate types. His scores were more comparable to COMPLEX cases and drastically different from Brad_M, yet his interview was also coded as MINIMAL. Although Carl_M described one of his voices as *an angry old man*, this was elicited from a direct question in the interview prompting Carl_M to describe the voice. More often, Carl_M described a narrow range of non-human entities (*it*, *banging*, *things*, *flies*, *shadows*). Carl_M did describe a capacity for the voices to physically affect him (*The banging can feel like, because it feels like that, then it hurts*) and attributed knowledge and a menacing motivation to them: *because it’s me, they know what to say, do you know, to annoy me*. He also referred to other intentional states that demonstrated agency, in that *sometimes they’ll just take, be other people’s voices and it will be their voice that it uses instead*. In this way, there was some indication of complexity – particularly in terms of independent thought and action – in Carl_M’s description of his voices, that was not apparent in, for example, Brad_M’s report. Nevertheless, this was limited in relation to the definition of complex personification. In fact, the report did not exhibit the features we have identified as characteristic of COMPLEX cases in Section 4.3. This supports his interview being coded as MINIMAL.

While, in each case, our analysis can be used to support the qualitative coding by Alderson-Day et al. (2020), the voices described by both Violet_C and Carl_M are complex in some respects and not in others. This suggests that, although they were treated differently in the binary coding, they are similar in that they both lie somewhere in the middle of a scale of personification that has Leah_C and Brad_M at opposite ends. In highlighting the more complex dimensions of each individual’s overall voice-hearing experience, we potentially identify areas for engagement with the voices that could inform personalised therapies. For instance, Violet_C appears to have positive encounters with *some* voices and negative encounters with others, suggesting that focusing on strategies to maximise the impact of the positive encounters and minimising the impact of the negative voices would have a positive effect overall. Similarly for Carl_M, the view that the voice has an agenda (i.e. to annoy Carl) that relies on Carl’s response, suggests that Carl has the capacity to shape the interaction, and that helping him with strategies to manage his response would be a productive goal for therapy.

## 6 Conclusions

In this paper, we have shown the contribution of corpus linguistic methods to an issue in clinical psychology that has so far been approached by means of qualitative coding: the ways in which voice-hearers describe their voices as persons, in the context of an intervention for people with psychosis. More specifically, our analysis of 40 semi-structured interviews that had been previously coded as involving minimal or complex personification offered an evaluation of the evidence to support such a coding, as well as developing that coding towards a scalar view, enriched with an understanding of the descriptive patterns associated with MINIMAL and COMPLEX cases.

The results of our direct comparison of COMPLEX and MINIMAL cases are convergent (Egbert & Baker, 2020b) with those of the qualitative coding scheme for complexity carried out by Alderson-Day et al. (2020):our approach provides quantitative linguistic evidence in support of the minimal/complex binary distinction at the group level. Furthermore, we showed that while the COMPLEX vs MINIMAL categories are valid, there is a significant amount of variation within the two categories, such that some MINIMAL interviews share characteristics with COMPLEX ones and vice versa. This was evident from the rank ordered lists of our interviews for each language component, but we were able to add further nuance to this point by examining what specific types make up the frequencies in different interviews for each language component. In this way, we were able to examine what specific types might be interpreted as indicative of complex personification. Finally, the rank-ordered lists of our interviews according to our language components provided us with an empirical basis on which to select individual interviews for further detailed analysis. We were able to focus on interviews that are respectively most and least prototypical of their complexity coding, and examine the degrees and types of personification that might place them in a particular position along a minimal-complex scale, but still explain their categorization.

In order to achieve these aims, and therefore perform a kind of triangulation that ismeaningful to/valid in clinical psychology, we had to start by operationalizing the notion of complex personification in terms that can be captured by corpus linguistic tools and manually annotating the data. We then had to adapt standard collocational and normalisation procedures in order to do justice to how language operates in our specific context. These adaptations can serve as examples to corpus linguists aiming to use corpus analysis for triangulation in highly specific social contexts. In this instance, we have provided data triangulation through using a range of methods (from psychology and from corpus linguistics) on the same dataset, as well as investigator triangulation in the researchers who carried out the separate analyses. Furthermore, our operationalisation of concepts as they are defined in clinical psychology (voice, personification, complexity) has also drawn on related concepts from linguistics, such as the grammatical realisation of agency and a view of personification informed by literary linguistics (Semino et al., 2021). This demonstrates one way in which qualitative coding approaches can be substantiated by quantitative and qualitative linguistic analysis.

From the point of view of clinical psychology our combined approach offers a way to validate case-study observations at the (complexity) group and population levels (via statistical tests). It also provides the measures that extend what was a binary classification to a scale, expanding the analysis towards degrees of complexity. Furthermore, the corpus approach provided an evidence base for authentic examples of the types (e.g. *want*, *make*, *different*) that were shown to be particularly meaningful in reporting complexity, since these were based on “real-life” reports from voice-hearers. While we would caution against the diagnostic use of such features, i.e. pointing to participants’ use of the particular types used by MINIMAL and COMPLEX participants as indicative of psychosis, they can provide the basis for monitoring progression in a (longitudinal) therapeutic context. For instance, one of the key features of complexity discussed here has been the reported agency of the voice and its capacity to affect the voice hearer. Tracking the quantity and quality of processes attributed to the voice(s) can provide insights into growing/diminishing agency on the part of the voice, which can be evaluated in comparison to attributions of agency to the self (i.e. the voice-hearer). Knapton (2021) has offered similar observations in relation to first-person accounts of Obsessive-Compulsive Disorder (OCD), arguing that the grammatical positioning of the self and the mind in participant reports can provide insights into their sense of agency, responsibility and blame that can direct therapists to areas of concern.

As the perception of voices as person has clinical and therapeutic implications, the use of corpusmethods we have exemplified in this paper is relevant both to the conceptualisation of personification in clinical psychology and to interventions aimed at enabling voice-hearers cope better with their voices.

## Figures and Tables

**Table 1:** Results of the statistical tests for comparisons of our language components between MINIMAL and COMPLEX participants

Language component	MINIMAL Mean	COMPLEX Mean	T-test	P-value	Effect size (Cohen’s *d*)	MINIMAL confidence intervals	COMPLEX confidence intervals
Voice label types	1.321	1.824	*t* (33.39)=-3.72	<.001	1.19	1.139–1.503	1.606–2.043
Adjective collocate types	0.814	1.174	*t* (25.27)=-3.20	<.01	1.10	0.695–0.932	0.967–1.380
Verb collocate types	2.165	2.853	*t* (28.67)=-3.57	<.01	1.19	1.938–2.393	2.516–3.189
Length (tokens)	3843.63	7170.31	*t* (18.14)=-3.19	<.01	1.20	3181.74–4505.51	5051.80–9288.83

**Table 2:** Rank lists for individual normalised frequencies for each language component

Participant	Voice label	Participant	Adj. collocates	Participant	Verb collocates	Participant	Tokens
1. Leah_C	2.63	1. Nina_C	1.78	1. Jade_C	3.96	1. Olivia_C	14475
2. Jade_C	2.55	2. Xander_C	1.76	2. Page_C	3.71	2. Dan_C	13852
3. Carl_M	2.48	3. Jane_C	1.61	3. Leah_C	3.54	3. Eric_C	13477
4. Olivia_C	2.17	4. Page_C	1.52	4. Jane_C	3.31	4. Hugh_C	10441
5. Zara_C	2.14	5. Grace_C	1.48	5. Xander_C	3.28	5. Leah_C	9647
6. Neil_M	2.09	6. Carl_M	1.38	6. Carl_M	3.22	6. Jade_C	7047
7. Sean_M	2.03	7. Neil_M	1.31	7. Zara_C	3.00	7. Page_C	6583
8. Kath_C	1.97	8. Kath_C	1.30	8. Emma_C	2.96	8. Zara_C	6334
9. Xander_C	1.95	9. Leah_C	1.24	9. Orla_C	2.93	9. Ryan_M	6222
10. Emma_C	1.86	10. Orla_C	1.23	10. Nina_C	2.92	10. Kate_M	5588
11. Page_C	1.85	11. Jade_C	1.18	11. Grace_C	2.87	11. Anthony_M	5553
12. Nina_C	1.82	12. Olivia_C	1.16	12. Iris_M	2.86	12. Mike_M	5435
13. Gail_M	1.79	13. Chris_M	1.08	13. Liam_M	2.83	13. Toby_M	5203
14. Grace_C	1.79	14. Bill_M	1.07	14. Sean_M	2.74	14. Alex_M	5047
15. Kate_M	1.57	15. Harry_M	1.06	15. Olivia_C	2.72	15. Xander_C	4977
16. Jane_C	1.56	16. Will_M	1.03	16. Neil_M	2.71	16. Yan_M	4931
17. Will_M	1.54	17. Iris_M	1.01	17. Yan_M	2.59	17. Kath_C	4783
18. Hugh_C	1.46	18. Ulrik_M	0.98	18. Matt_M	2.50	18. Violet_C	4782
19. Dan_C	1.43	19. Liam_M	0.97	19. Mike_M	2.45	19. Emma_C	4688
20. Orla_C	1.42	20. Fran_M	0.94	20. Gail_M	2.37	20. Dawn_M	4681
21. Toby_M	1.38	21. Ryan_M	0.93	21. Kath_C	2.36	21. Sean_M	4676
22. Eric_C	1.37	22. Dan_C	0.82	22. Bill_M	2.28	22. Will_M	4515
23. Fred_M	1.34	23. Emma_C	0.82	23. Ryan_M	2.23	23. Harry_M	4396
24. Anthony_M	1.33	24. Eric_C	0.80	24. Hugh_C	2.20	24. Fran_M	4260
25. Liam_M	1.32	25. Sean_M	0.78	25. Alex_M	2.19	25. Neil_M	4238
26. Ian_M	1.25	26. Gail_M	0.77	26. Harry_M	2.15	26. Fred_M	4180
27. Matt_M	1.23	27. Zara_C	0.77	27. Chris_M	2.13	27. Nina_C	4111
28. Yan_M	1.23	28. Violet_C	0.76	28. Eric_C	2.09	28. Iris_M	4029
29. Violet_C	1.22	29. Toby_M	0.74	29. Ian_M	2.08	29. Jane_C	3988
30. Mike_M	1.18	30. Yan_M	0.73	30. Will_M	2.08	30. Brad_M	3862
31. Brad_M	1.17	31. Mike_M	0.71	31. Ulrik_M	1.95	31. Bill_M	3762
32. Iris_M	1.17	32. Alex_M	0.60	32. Toby_M	1.94	32. Orla_C	3418
33. Bill_M	1.12	33. Anthony_M	0.59	33. Dan_C	1.91	33. Chris_M	2666
34. Harry_M	1.11	34. Ian_M	0.59	34. Violet_C	1.88	34. Gail_M	2143
35. Ulrik_M	1.06	35. Fred_M	0.58	35. Fran_M	1.73	35. Grace_C	2122
36. Alex_M	1.02	36. Hugh_C	0.55	36. Kate_M	1.62	36. Liam_M	1943
37. Ryan_M	0.99	37. Matt_M	0.54	37. Dawn_M	1.57	37. Ian_M	1296
38. Chris_M	0.98	38. Brad_M	0.45	38. Anthony_M	1.50	38. Carl_M	1259
39. Fran_M	0.85	39. Dawn_M	0.36	39. Brad_M	1.14	39. Matt_M	1224
40. Dawn_M	0.47	40. Kate_M	0.33	40. Fred_M	1.11	40. Ulrik_M	1138

## Footnotes

1 Participants have been given pseudonyms to protect their identities.

2 We use the capitalised notation to refer to the specific classification of the interview data according to the coding scheme applied by the Hearing the Voice team.

3 See Resnik et al. (2014) for a discussion of the more general use of computational linguistics in the context of clinical psychology.

4 Computational approaches to this issue of ‘coreference resolution’ developed in Natural Language Processing research have typically relied on knowledge-rich algorithms, tailored to particular types of text (for an overview, see Sukthanker et al., 2020). We favoured a manual annotation of this relatively small (i.e. manageable) corpus, to ensure an exhaustive account of references to ‘voices’.

5 In the sense that participants with more COMPLEX experiences might have more to report and describe.

6 *#LancsBox* automatically tags texts for parts-of-speech and recognises any input following an underscore as annotation (e.g. _VOICE) as a user-defined tag.

7 According to Cohen (1988), d=0.2 is considered a ‘small’ effect size, 0.5 represents a ‘medium’ effect size and 0.8 a ‘large’ effect size.

8 That is not to say that there were no other indicators of gender in MINIMAL cases. Gender could still be indicated in the use of pronouns (she, he) and particular proper names (*Roxy*, *David*).
